# A new giant *Pristimantis* (Anura, Craugastoridae) from the paramos of the Podocarpus National Park, southern Ecuador

**DOI:** 10.3897/zookeys.852.24557

**Published:** 2019-06-05

**Authors:** Mario H. Yánez-Muñoz, David Veintimilla-Yánez, Diego Batallas, Diego F. Cisneros-Heredia

**Affiliations:** 1 Instituto Nacional de Biodiversidad, Unidad de Investigación, Casilla 17-07-8976, Quito, Ecuador Unidad de Investigación Quito Ecuador; 2 Universidad Nacional de Loja, Loja, Ecuador Pontificia Universidad Católica del Ecuador Quito Ecuador; 3 Universidad San Francisco de Quito USFQ, Colegio de Ciencias Biológicas y Ambientales, Laboratorio de Zoología Terrestre y Museo de Zoología, Quito 170901, Ecuador Universidad Nacional de Loja Loja Ecuador; 4 Pontificia Universidad Católica del Ecuador, Escuela de Biología, Museo de Zoología QCAZ, Quito, Ecuador Ministerio del Ambiente, Dirección Nacional de Biodiversidad Quito Ecuador; 5 King’s College London, Department of Geography, Strand, London, UK Universidad San Francisco de Quito USFQ Quito Ecuador; 6 Ministerio del Ambiente, Dirección Nacional de Biodiversidad, Quito, Ecuador King’s College London London United Kingdom

**Keywords:** Amphibia, Andes, Cajanuma, Craugastoridae, Loja, new species, paramo, *
Pristimantis
*, taxonomy, Zamora-Chinchipe, Amphibia, Andes, Cajanuma, Craugastoridae, Loja, nueva especie, páramo, *
Pristimantis
*, taxonomía, Zamora-Chinchipe

## Abstract

A new species of frog of the genus *Pristimantis* is described from the paramos of the Nudo de Cajanuma, Podocarpus National Park, on the border between the provinces of Loja and Zamora-Chinchipe, Ecuador. The new species is readily distinguished from all other species of *Pristimantis* by its large body size (snout-vent length: 50.0–50.5 mm in adult females, 34.7–42.5 mm in adult males), thick glandular skin, large warts on flanks, prominent glandular patches on head and legs, and dark brown dorsum. This new species is among the largest and stoutest *Pristimantis* frogs of the high Andes. It is only known from its type locality, where it occurs in paramo bambusoid meadows at elevations between 3300 and 3400 m. It is morphologically similar to *Pristimantiserythros*, *P.farisorum*, *P.obmutescens*, *P.orcesi*, *P.racemus*, *P.simoterus*, *P.simoteriscus*, and *P.thymelensis*. Notorious morphological characters present in this new species are thick glandular patches covering dorsum and limbs and porous skin texture, which are shared with *P.erythros*.

## Abstract (es)

Describimos una nueva especie de rana del género *Pristimantis* de los páramos del Nudo de Cajanuma, Parque Nacional Podocarpus, en el límite entre las provincias de Loja y Zamora-Chinchipe. La nueva especie se diferencia de otras especies de *Pristimantis* por su gran tamaño corporal (longitud rostro-cloacal: 50,0–50,5 mm en hembras adultas, 34,7–42,5 mm en machos adultos), piel glandular y gruesa, verrugas grandes en los flancos del cuerpo, prominentes parches glandulares en la cabeza y patas, y dorso café oscuro. Esta nueva especie está entre las ranas *Pristimantis* más grandes y fornidas de los altos Andes. Solo se conoce de su localidad tipo, donde habita en herbazales bambusoides de páramo a elevaciones entre 3300 y 3400 m. Es morfológicamente similar a *Pristimantiserythros*, *P.farisorum*, *P.obmutescens*, *P.orcesi*, *P.racemus*, *P.simoterus*, *P.simoteriscus*, y *P.thymelensis*. Características morfológicas notorias en esta nueva especie son los parches glandulares gruesos que cubren el dorso y las patas y la textura de la piel porosa, las cuales son compartidas con *P.erythros*.

## Introduction

*Pristimantis* (Jiménez de la Espada 1870) is the most diverse amphibian genus, representing nearly 8% (532 spp.) of all named anuran species worldwide (Frost 2019). Endemic to tropical America, *Pristimantis* is distributed from Honduras to Argentina, and is the most-species rich genus in anuran communities of the tropical Andes, from Colombia to Bolivia. Frogs of the genus *Pristimantis* show vast morphological and ecological diversity, which is associated with a complex and challenging taxonomy (Duellman and Lehr 2009, Pinto-Sánchez et al. 2012, Padial et al. 2014). Ecuador holds 39% of all *Pristimantis* species (210 spp., Ron et al. 2019), but its diversity is still underestimated, and 61 species have been discovered and scientifically described in the country during the last decade (Ron et al. 2019). *Pristimantis* from the Andes of southern Ecuador are little known; with new species frequently discovered, new distributional records revealed, collections of putatively new species deposited in museum collections, and several areas unexplored (Bustamante and Mendelson III 2008, Cisneros-Heredia et al. 2009, Reyes-Puig et al. 2010, 2010, 2014, 2015, Yánez-Muñoz et al. 2010, 2010, 2010, 2012, 2016, 2016, 2016, Camacho-Badani et al. 2012, Reyes-Puig and Yánez-Muñoz 2012, Brito and Pozo-Zamora 2013, Urgilés et al. 2014, 2014, 2014, Brito et al. 2017, Urgiles et al. 2017, Sánchez-Nivicela et al. 2018, Reyes-Puig et al. 2019).

The Podocarpus National Park is located on the southernmost portion of the Cordillera Oriental of the Andes, in the provinces of Loja and Zamora-Chinchipe, southern Ecuador. It protects about 1450 km^2^ from 900 to 3600 m elevation, including foothill, low montane, cloud, high montane forests and paramos (MAE 2017). Little information exists about the herpetofauna of the highlands of Podocarpus National Park. Between 2009 and 2010, herpetological surveys were conducted on the paramos of Cajanuma, western side of the Podocarpus National Park, from 3320 to 3365 m elevation, as part of a project to evaluate the impacts of climate change on the biodiversity of this ecosystem (Salinas Salinas and Veintimilla-Yánez 2010, L Aguirre Mendoza et al. 2015). During these surveys, three putatively new species of *Pristimantis* were collected. Herein, we are pleased to describe and name one of these species.

## Materials and methods

Field work was carried out between December 2009 and April 2010 in the paramos of the Nudo de Cajanuma (nudo is the local name for transverse mountain ranges), Podocarpus National Park, on the border between the provinces of Loja and Zamora-Chinchipe, Ecuador. Paramos are highland Neotropical ecosystems dominated by grasses and forbs and located between the forest upper limit and the permanent snow line in the Andes from Venezuela to northern Peru (Acosta-Solís 1984, Luteyn 1999). While most paramos occur above 3400 m elevation, in southern Ecuador paramos are found from 2080 m elevation due to local climate and geology (Neill 1999, León-Yánez 2011). The physiography of the paramo of Cajanuma is characterised by series of hills with steep slopes, connected by ridges, and dissected by small streams. While paramos in the Podocarpus National Park typically receive an average annual precipitation < 5000 mm, the paramo of Cajanuma is wetter and may receive up to 6000 mm. During most of the year, local weather is characterized by persistent cloud cover, fierce easterly winds, and low temperatures (maximum daily temperature ≈ 10° C, minimum typically between 0–3 °C), although a short dry season may occur during November and December (Keating 1999, 2008, Lozano et al. 2009, Aguirre et al. 2015). Vegetation is characterised by a diverse physiognomy of grasses, forbs, shrubs, and treelets. Descriptions of the flora of the paramo of Cajanuma were provided by Keating (1999, 2008) and Eguiguren et al. (2015).

Herpetological surveys were conducted at the paramo of Cajanuma across an area located at the following coordinates: 79.16219444°–79.16111111°W, 4.10861111°–4.09466667°S, at 3320–3365 m elevation (Salinas Salinas and Veintimilla-Yánez 2010). Coordinates were obtained by means of a Garmin Handheld Navigator GPS (WGS84). Two survey techniques were used: visual encounters during evenings (19:00–22:00) and rake and hoe removal of plant rosettes during mornings (09h00–12h00) (Mueses-Cisneros and Yánez-Muñoz 2009, Heyer et al. 2014). Specimens were photographed alive, euthanised with benzocaine, fixed in 10% formalin, and preserved in 70% ethanol.

**Figure 1.**
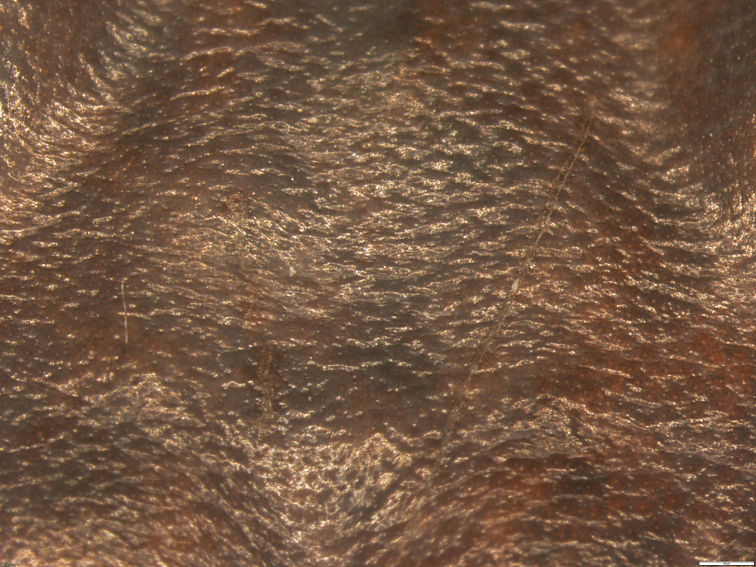
Porous texture of the skin on the middorsum of *Pristimantisandinogigas* sp. nov. (DHMECN 11013, adult male, paratype).

Description format, definitions and terminology follows standards proposed by Lynch and Duellman (1997) and Duellman and Lehr (2009). For skin texture, we include a new descriptor: porous skin, which is defined by showing small pores evenly distributed across the skin. Areolate, pustulate and shagreen skin textures differ from porous skin by having dermal modifications (granules, protuberances) raised from the background plane of the skin, while the pores of porous skin are below the background plane (Fig. 1; compare with Duellman and Lehr 2009: fig. 39). Sex and age were determined by direct inspection of gonads. The following measurements were taken with digital calipers to the nearest 0.01 mm and rounded to the nearest 0.1 mm by a single person (David Veintimilla-Yánez): snout-vent length (SVL), straight distance from tip of snout to vent; head width, at angle of jaws; head length, from angle of jaw to tip of snout; eye diameter, horizontally from anterior to posterior corner of eye; interorbital distance, shortest distance between orbits; internarial distance, shortest distance between nostrils; eye-nostril distance, straight distance between anterior corner of eye and posterior margin of nostril; tympanum diameter, greatest horizontal width of tympanum; tibia length, distance from outer border of flexed knee to heel inflection; hand length, distance from base of tenar tubercle to tip of Finger III; and foot length, distance from base of inner metatarsal tubercle to tip of Toe IV. Fingers and toes are numbered preaxially to postaxially from I to IV and I to V, respectively. Comparative lengths of Toes III and V were determined by adpressing both against Toe IV; lengths of Fingers I and II were compared when adpressed against each other. Photographs and field notes were used for descriptions of colouration in life.

Examined specimens are deposited at: División de Herpetología, Instituto Nacional de Biodiversidad, Quito (**DHMECN**); Instituto de Ciencias Naturales, Universidad Nacional de Colombia, Bogotá (**ICN**); Laboratorio de Herpetología, Universidad del Valle, Cali (**UVC**), and Museo de Zoología, Universidad San Francisco de Quito (**ZSFQ**). Information on species for comparative diagnoses was obtained from examined specimens and literature, including original species descriptions. The following specimens were examined for comparisons:

**ECUADOR**: *Pristimantiserythros*: Provincia del Azuay: Chanlud, 3449 m, DHMECN 12103, holotype, DHMECN 12102, paratype, ZSFQ 034–036. *Pristimantisloujosti*: Provincia de Tungurahua: Reserva Biológica La Candelaria, 2800 m, DHMECN 4843. *Pristimantisorcesi*: Provincia de Napo: Cuyuja, 3591 m, DHMECN 2903. *Pristimantisthymelensis*: Provincia de Carchi: Reserva Ecológica El Ángel, 3900 m, DHMECN 1867-1890; Lagunas del Voladero, 3420 m, DHMECN 2415-2419; Provincia de Napo: Papallacta, 3910 m, DHMECN 1856. **COLOMBIA**: *Pristimantisobmutescens*: Departamento del Cauca: Páramo de Puracé, alrededores de la Laguna San Rafael, ICN 2087, holotype. *Pristimantisracemus*: Departamento del Valle del Cauca, Tenerife, UVC 8131, 8193. *Pristimantissimoteriscus*: Departamento del Tolima: Municipio de Cajamarca, Páramo de los Valles, SW of Anaime, Anaime-Santa Helena road, ICN 22835, holotype. *Pristimantissimoterus*: Departamento del Tolima: Páramo de Letras, vereda Albania, Municipio de Herveo, ICN 759, holotype.

Vocalizations were recorded with an Olympus WS-750 digital recorder and a Senheiser K6-C unidirectional microphone. During the recordings, air temperature and relative humidity were measured with a Springfield environmental thermometer. Acoustic analyses were done with Adobe Audition 3.0 software package (Adobe Systems Inc., San Jose, California, USA), at a sampling frequency of 44.1 kHz and 16-bit resolution. Waveform and spectrogram were made using Raven Pro 1.4 software package (Cornell Lab of Ornithology, Ithaca, NY) and analysed with a Fast Fourier Transformation of 512 points. Terminology and definitions follow proposals by Duellman and Pyles (1983), Cocroft and Ryan (1995), Díaz and Cádiz (2006), and the following variables were measured: (1) dominant frequency of the call, (2) dominant frequency of the first note, (3) dominant frequency of the second note, (4) calls per minute, (5) notes per call, (6) call duration, (7) note duration, (8) interval between calls, and (9) intervals between notes within a call.

## Results

### Pristimantis andinogigas sp. nov.

Taxon classificationAnimaliaAnuraCraugastoridae

http://zoobank.org/3BF7D08B-5586-4314-AC76-B40D724C1F97

**Figure 2.**
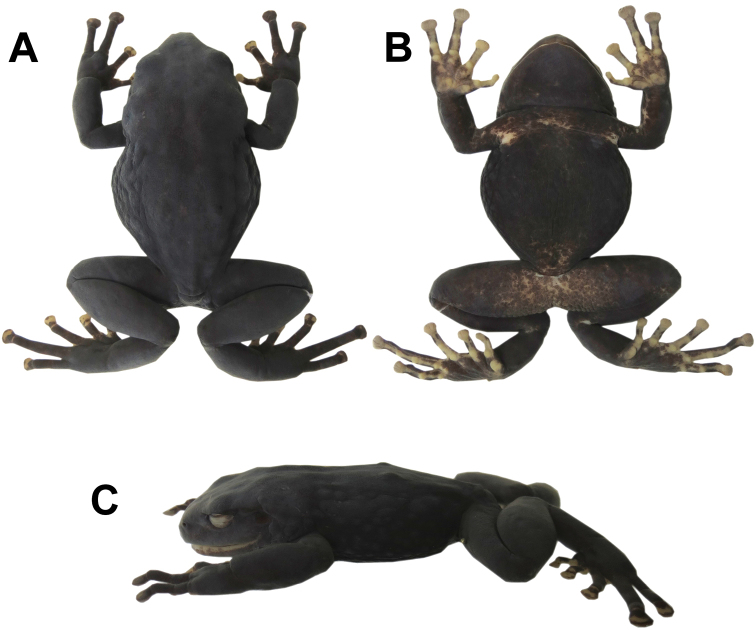
Holotype of *Pristimantisandinogigas* sp. nov. (DHMECN 10984, adult female, 50.0 mm snout-vent length) in dorsal (**A**) ventral (**B**) and lateral (**C**) views of preserved specimen.

**Figure 3.**
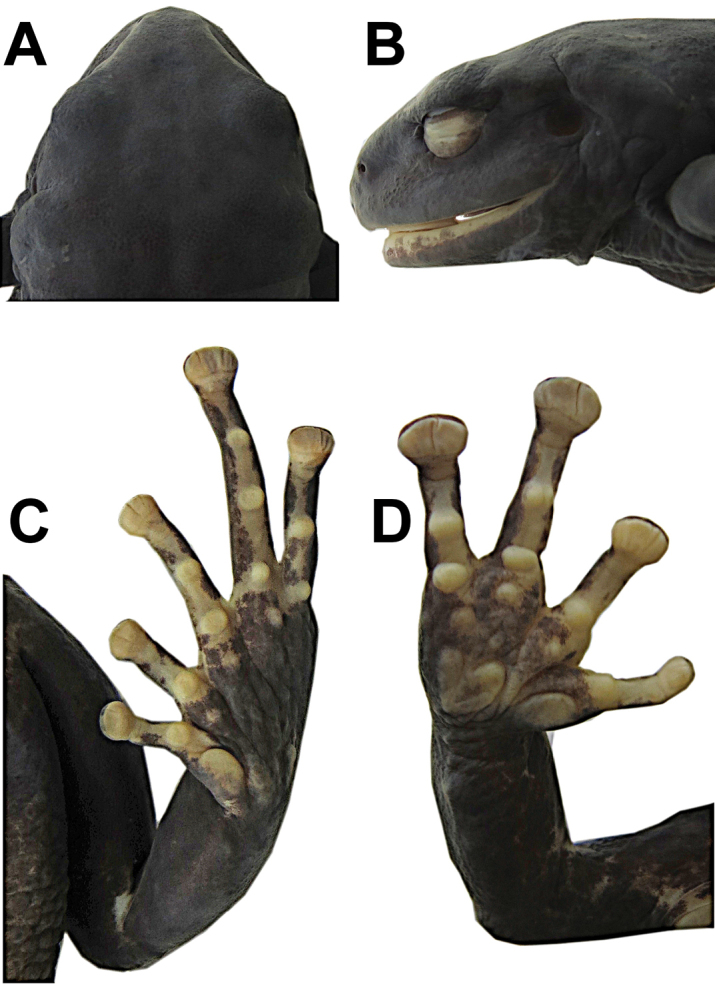
Dorsal (**A**) and lateral (**B**) views of head, and ventral views of hand (**C**) and foot (**D**) of holotype of *Pristimantisandinogigas* sp. nov. (DHMECN 10984, adult female, head length 16.0 mm, head width 20.1 mm, hand length 15.8 mm, foot length 24.3 mm).

**Figure 4.**
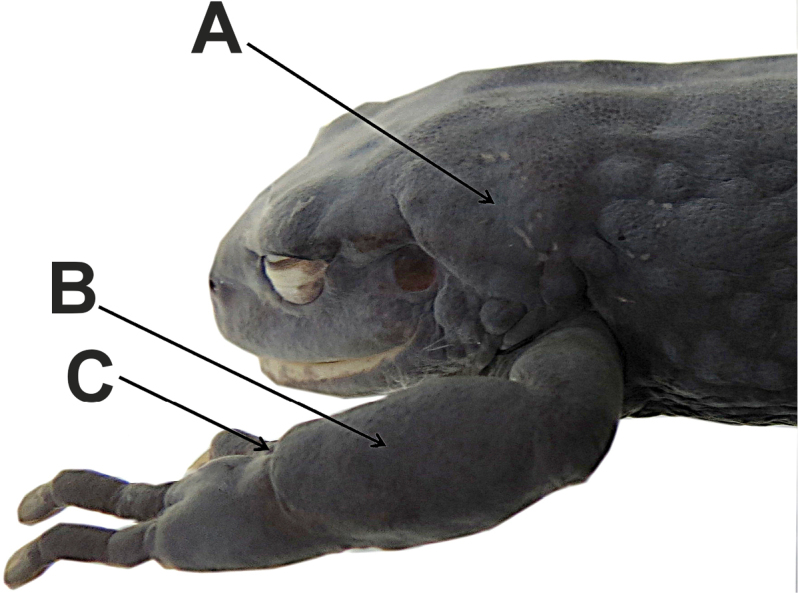
Dorsolateral view of head of holotype of *Pristimantisandinogigas* sp. nov. (DHMECN 10984, adult female) showing supratympanic (**A**) and humeral (**B**) glandular patches, and glandular fold of wrist (**C**). Note porous dorsal skin texture.

**Figure 5.**
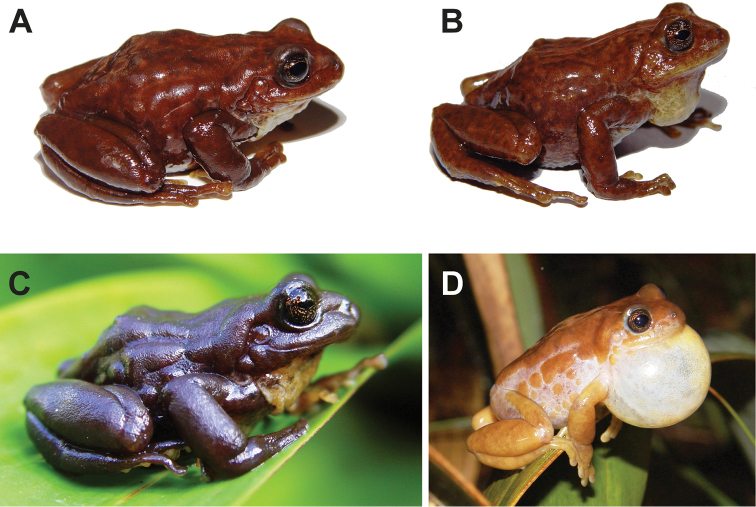
Dorsolateral view of three live male paratypes (**A–B, D**) and live female holotype (**C**) of *Pristimantisandinogigas* sp. nov.: DHMECN 10985 (**A**) DHMECN 10993 (**B**) DHMECN 10984 (**C**) DHMECN 11000 (**D**). Photographs by David Veintimilla-Yánez.

Figures 1 , 2 , 3 , 4 , 5

Pristimantis grp. orcesi: L Aguirre Mendoza et al. 2015: 173, 180; Z Aguirre Mendoza et al. 2017: 534–535.

#### Common names.

English: Giant paramo rainfrog. Spanish: Cutín Gigante de Páramo.

#### Holotype.

Adult female; ECUADOR; provincia de Loja, Parque Nacional Podocarpus, Cajanuma; 4.108346°S, 79.162046°W, 3313 m alt.; 27 January 2010; David Veintimilla-Yánez and Karen Salinas leg.; DHMECN 10984 (field number DVY 057).

#### Paratypes.

Same collection data as for holotype; DHMECN 10985–6, adult males, 09 December 2009; DHMECN 10996, adult female, and DHMECN 10991–2, adult males, 10 December 2009; DHMECN 10993–4, adult males, 06 January 2010; DHMECN 10998–9, adult males, 06 January 2010; DHMECN 11000, adult male, 07 January 2010; DHMECN 11005, adult male, 13 January 2010; DHMECN 11008, adult male, 14 January 2010; DHMECN 11010–1, adult males, 27 January 2010; DHMECN 11012–13, 11115, adult males, 31 March 2010; DHMECN 11016, adult male, 06 April 2010; subadult males: DHMECN 10997, 06 January 2010; DHMECN 11001, 07 January 2010; subadult females: DHMECN 10995, 06 January 2010; DHMECN 11002, 07 January 2010; DHMECN 11006, 13 January 2010; DHMECN 11007, 14 January 2010; DHMECN 11018, 12 April 2010; DHMECN 11021, 22 April 2010; juveniles: DHMECN 10987–8, 10990, 09 December 2009; DHMECN 10989, 10 December 2009; DHMECN 11003–4, 13 January 2010; DHMECN 11009, 26 January 2010; DHMECN 11014, 31 March 2010; DHMECN 11017, 07 April 2010; DHMECN 11019, 14 April 2010; DHMECN 11020, 19 April 2010.

#### Diagnosis.

A new species of *Pristimantis* diagnosed by the following combination of characters: (1) Skin on dorsum porous, thick and glandular, with large, flat, glandular warts on flanks; dorsolateral folds absent; thick glandular patch on supra/postympanic region, and on dorsal surfaces of humeral, femoral, tibial and tarsal regions; glandular folds in wrists; skin on venter areolate; discoidal fold weakly defined; (2) tympanic membrane and tympanic annulus prominent; tympanic annulus rounded, 36% of eye length, with posterior margin in contact with supratympatic glandular patch; (3) snout rounded in dorsal view; rounded to slightly protruding in lateral view; (4) upper eyelid without tubercles, IOD wider than upper eyelid; cranial crests absent; (5) dentigerous processes of vomers present, oblique, moderately separated, posteromedial to choanae, with 4 to 5 teeth; (6) males with cream-coloured nuptial pads on dorsum of Finger I and vocal slits; (7) Finger I shorter than Finger II; emarginated discs of fingers broadly expanded and elliptical; (8) fingers without lateral fringes; (9) ulnar tubercle present but low or poorly differentiated; (10) heels without tubercles, inner tarsal wart low and poorly differentiated; (11) inner metatarsal tubercle ovoid, about 5–6x the size of subconical, rounded outer metatarsal tubercle; supernumerary plantar tubercles present; (12) toes with narrow lateral fringes; basal toe webbing between toes II–V; Toe V longer than Toe III (disc of Toe III does not reach distal subarticular tubercle on Toe IV, disc on Toe V reaches middle of distal subarticular tubercle on Toe IV); toe discs elliptical, slightly narrower than those on fingers; (13) in life, dorsal surfaces dark brown, chocolate brown, or orange-brown, with or without dark irregular botches, distinctive head markings absent, ventral surfaces brown with irregular pale flecks and blotches, iris bronze with dense black reticulations; in preservative, brown surfaces turn grey; (14) SVL 50.0–50.5 mm in adult females (*n* = 2), 34.7–42.5 (38.5 ± 2.1 SD, *n* = 10) mm in adult males (Table 1).

**Table 1.** Measurements (in mm) of type series of *Pristimantisandinogigas* sp. nov. from Nudo de Cajanuma, Podocarpus National Park, Andes of southern Ecuador. For males, range is followed by means and one standard deviation in parentheses.

**Characters**	**Females (*n* = 2)**	**Males (*n* = 17)**
Snout-vent length	50.0–50.5	34.7–42.5 (38.5 ± 2.1)
Head width	19.3–20.1	13.3–15. 9 (14.6 ± 0.8)
Head length	16.0–17.4	11.2–13.9 (12.7 ± 0.72)
Eye diameter	5.6–6.0	4.9–5.6 (5.3 ± 0.2)
Interorbital distance	8.2–8.8	5.5–7.9 (6.1 ± 0.6)
Internarial distance	4.4–4.6	3.5–4.6 (4.0 ± 0.3)
Eye-nostril distance	4.9–5.3	3.8–4.9 (4.1 ± 0.3)
Tympanum diameter	2.1–2.7	1.5–2.1 (1.8 ± 0.2)
Tibia length	22.1–23.2	16.5–19.0 (17.9 ± 0.7)
Hand length	15.7–15.8	11.0–13.3 (12.0 ± 0.7)
Foot length	23.7–24.3	16.3–20.4 (18.2 ± 1.0)

#### Comparisons.

*Pristimantisandinogigas* sp. nov. is readily distinguished from all other species of *Pristimantis* by its large body size, thick and glandular skin, large warts on flanks, prominent macroglandular patches on head and legs, and dark brown dorsum. The only species showing a similar combination of characters is *Pristimantiserythros* Sánchez-Nivicela, Celi-Piedra, Posse-Sarmiento, Urgiles, Yánez-Muñoz & Cisneros-Heredía, 2019, which is readily differentiated from *P.andinogigas* sp. nov. by being smaller (38.8–42.6 mm in adult females), having a conspicuous red coloration, and lacking dentigerous processes of vomers. In addition, *P.andinogigas* sp. nov. resembles the following species by bearing large, flat, glandular warts on flanks, and expanded discs on fingers and toes: *Pristimantisfarisorum* Mueses-Cisneros, Perdomo-Castillo, & Cepeda-Quilindo, 2013, *P.obmutescens* (Lynch, 1980), *P.orcesi* (Lynch, 1972), *P.racemus* (Lynch, 1980), *P.simoterus* (Lynch, 1980), *P.simoteriscus* (Lynch), and *P.thymelensis* (Lynch, 1972). *Pristimantisandinogigas* sp. nov. is larger than any of these seven species, and furthermore, they differ from *P.andinogigas* as follows (characters of *P.andinogigas* sp. nov. in parentheses): areolate or shagreen dorsal skin (porous), thin supratympanic folds (prominent supra/post-tympanic glandular patch), thin glandular patches on legs (thick), and smaller body size, with adult females having 38.4–42.3 mm SVL in *P.farisorum*, 28.5–38.4 mm SVL in *P.obmutescens*, 35.2–36.1 mm SVL in *P.orcesi*, 29.9–37.9 mm SVL in *P.racemus*, 32.4–37.1 mm SVL in *P.simoterus*, 25.7–31.4 mm SVL in *P.simoteriscus*, and 28.0–33.5 mm SVL in *P.thymelensis* (versus 50.0–50.5 mm SVL in adult females of *P.andinogigas*). In addition, *P.farisorum* has snout subacuminate in dorsal view (rounded), fingers with narrow lateral fringes (absent), dorsum dark brown to black with irregular and elongated orange marking (brown with or without lighter irregular blotches), and inhabits upper montane forests on the Nudo de Pasto, Andes of southern Colombia (Mueses-Cisneros et al. 2013). *Pristimantisobmutescens* has tympanum concealed beneath skin (visible), fingers with lateral fringes present (absent), small, non-conical tubercles on heel and outer edge of tarsus present (absent), lacks vocal sac and vocal slits in males (present), and inhabits on the Páramo de Puracé, Cordillera Central of the Andes in southern Colombia (Lynch 1980, Lynch et al. 1996). *Pristimantisorcesi* has skin on head smooth (porous), fingers bearing lateral fringes (absent), lacks dentigerous processses of vomers (present), and inhabits paramos on the Andes of north-central Ecuador (Lynch 1972, 1981). *Pristimantisracemus* has fingers with lateral fringes (absent), dorsum reddish-brown with darker marking (brown with or without darker irregular blotches), lacks vocal sac and vocal slits in males (present), and inhabits paramos on the Cordillera Central of the Andes, central Colombia (Lynch 1980, Lynch et al. 1996). *Pristimantissimoterus* has fingers with lateral fringes (absent) and inhabits upper montane forests and paramos on the Cordillera Central of the Andes, central Colombia (Lynch 1980, Lynch et al. 1996). *Pristimantissimoteriscus* has subacuminate snout in dorsal view, fingers with lateral fringes (absent), dorsum grey with dark markings (brown with or without darker irregular blotches), lacks vocal slits in males, and inhabits paramos on the Cordillera Central of the Andes, central Colombia (Lynch et al. 1996). *Pristimantisthymelensis* has tympanum concealed beneath skin (visible), paraventral folds present (absent), finger bearing lateral fringes (absent), grey to brown dorsum speckled to varying degrees with creamy grey, tan, or black (brown with or without darker irregular blotches), and inhabits paramos on Andes of southern Colombia and northern Ecuador (Lynch 1972, 1981).

*Pristimantisloujosti* and *P.pycnodermis* also stand out from other species of the genus by their stout body and thick glandular skin on dorsal surfaces of body and limbs, but they differ from *P.andinogigas* sp. nov. as follows (characters of *P.andinogigas* sp. nov. in parentheses): *Pristimantisloujosti* Yánez-Muñoz, Cisneros-Heredia & Reyes-Puig, 2010 has smooth skin on head and granular skin on dorsum and flanks (porous, with large warts on flanks), thick supratympanic fold (prominent glandular supra/post-tympanic patch), thin glandular patches on legs (thick), subacuminate snout in dorsal view (rounded in dorsal view), fingers bear lateral fringes (absent), black spots on hidden surfaces of limbs (uniformly coloured), light iris with dark reticulation (bronze with dense black reticulations), and it inhabits on cloud forests on the Upper River Pastaza, Cordillera Oriental of the Andes of Ecuador (Yánez-Muñoz et al. 2010). *Pristimantispycnodermis* (Lynch, 1979) differs by having low cranial crests (absent), snout subacuminate in dorsal view and truncate in lateral view (snout rounded in dorsal view; rounded to slightly protruding in lateral view), skin of flanks smooth (with large warts), fingers bear lateral fringes (absent), dark canthal and tympanic marks (head marks absent), large black spots on the flanks (brown with or without dark irregular blotches), 32.5–44.4 mm SVL in adult females (50.0–50.5 mm), and inhabits paramos on the Andes of central-southern Ecuador (Lynch 1979).

#### Description of holotype.

Adult female (50.0 mm SVL, Fig. 2); head narrower than body, wider than long (head width 40% of SVL, head length 32% of SVL, head length 80% of head width); snout short (eye nostril 11% of SVL, eye nostril 87% of eye diameter), rounded in dorsal and lateral views; canthus rostralis rounded and weakly concave; loreal area concave; lips flared; eye large (eye diameter 1.14 times eye-nostril distance, eye diameter 38% of head length); nostrils slightly protuberant laterally (Fig. 3). Cranial crest absent; upper eyelids without tubercles; tympanic membrane differentiated, tympanic annulus visible (tympanum diameter 35% of eye diameter), upper and posterior borders of tympanic annulus in contact with prominent, thick glandular patch that covers all dorsal fascia of m. *depressor mandibulae*; large, glandular postrictal tubercles. Choanas small and widely separated from each other, not concealed by palatal shelf of maxilla; dentigerous processes of vomer present, oblique, moderately separated, posteromedial to choanae, with four or five teeth; tongue longer than wide, posterior half not adherent to floor of mouth.

Skin on dorsum thick and glandular, surface texture porous (Figs 1, 4), with large, flat, glandular warts on flanks; dorsolateral folds absent; thick glandular patch on dorsal surfaces of humeral, femoral, tibial and tarsal regions; glandular folds in wrists (Fig. 4); skin on venter areolate; discoidal fold weakly defined; skin on ventral surfaces of legs granular; cloaca not protuberant, cloacal region with large warts. Ulnar tubercle present but low; palmar tubercle flat and bifurcate; thenar tubercle elongate, about half the size of palmar tubercle; subarticular tubercles prominent, rounded in ventral and lateral views; supernumerary palmar tubercles rounded, smaller than subarticular tubercles; fingers without lateral fringes; Finger I shorter than Finger II; discs on fingers expanded and elliptical, most prominent on fingers II–IV, while disc on Finger I slightly expanded; all discs bearing ventral pads well defined by circumferential grooves (Fig. 3).

Hind limbs robust (tibia length 46% of SVL; foot length 49% of SVL); heel without tubercles; inner edge of tarsus with one wart low and poorly differentiated; inner metatarsal tubercle ovoid, about 5x round outer metatarsal tubercle; subarticular tubercles rounded; plantar supernumerary tubercles low and inconspicuous, smaller than subarticular tubercles; toes with narrow lateral fringes; basal toe webbing between toes II–V; discs of toes expanded, elliptical, slightly narrower than those on fingers, most prominent on fingers II–V, while disc on Finger I slightly expanded; toes with ventral pads well-defined by circumferential grooves; toe lengths, when adpressed, IV > V > III > II > I; Toe V longer than Toe III; disc of Toe III not reaching distal subarticular tubercle on Toe IV, disc on Toe V reaches middle of distal subarticular tubercle on Toe IV (Fig. 3).

#### Measurements (in mm) of holotype.

Snout-vent length 50.0; head width 20.1; head length 16.0; eye-nostril distance 5.3; internarial distance 4.6; interorbital distance 8.8; tympanum diameter 2.1; eye diameter 6.0; tibia length 23.2; hand length 15.8; foot length 24.3.

#### Colouration of holotype in life.

Dorsum dark brown; ventral surfaces dark brown with irregular light-yellow flecks and blotches on throat, hands, feet, armpits, and lower venter; iris golden-bronze with dense black reticulations (Fig. 5).

#### Colouration of holotype in preservative.

Same pattern as in life, but brown surfaces turned dark grey (Fig. 2).

#### Variation.

Males are smaller than females, measurements of the type series are provided in Table 1. Dorsal colouration of body and legs varies from dark brown, chocolate brown, or orange-brown (Fig. 5.). Females are darker and have a homogeneous coloration pattern, while males are paler and usually with dark irregular blotches. Some individuals have pale pink flanks and dorsal surfaces of legs (Fig. 5D). Venter colouration varies from completely dark brown to dark brown with irregular light-yellow flecks and blotches. Background dorsal colouration of juveniles is paler, and dorsal dark blotches are darker.

#### Etymology.

The specific epithet is coined from the New Latin adjective *andinus* (pertaining to the Andes) and the Latin noun *gigas* (giant). The name alludes to the large and stout body of this new species in comparison with other species of *Pristimantis* from the high Andes.

#### Vocalizations.

**Figure 6.**
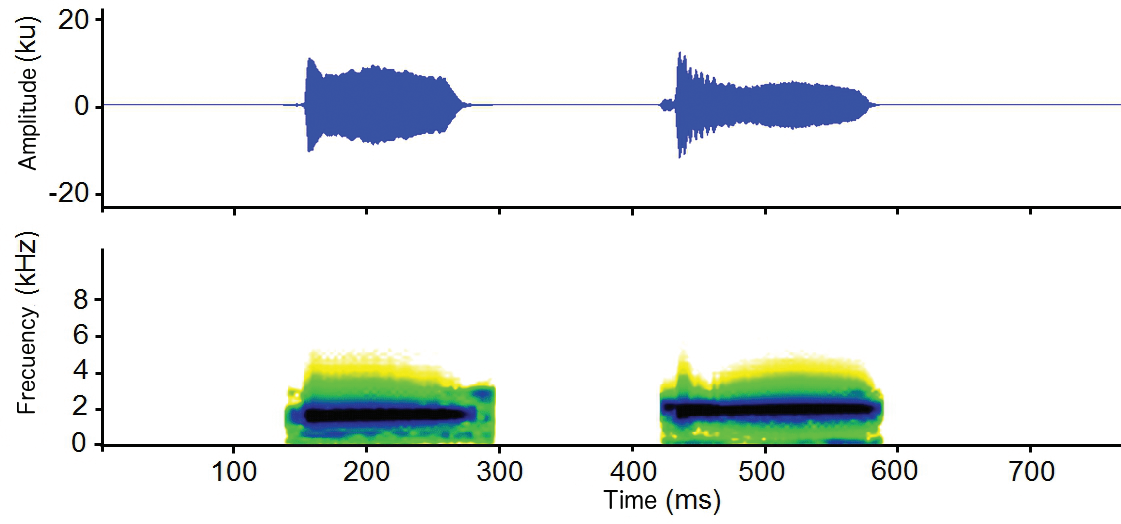
Advertisement call of *Pristimantisandinogigas* sp. nov. (paratype, DHMECN 11016).

Males call from grasses at night, in heterogeneous chorus with extensive call superposition. Paratype DHMECN 11016 was calling from bamboos *Neurolepis* sp. (T_air_ = 7° C, relative humidity = 96%). The advertisement call (Fig. 6) has dominant frequencies of 1.63–1.98 kHz (1.80 ± 0.14 SD kHz). Calls were 124–428 ms (231.37 ± 142.76 ms) in duration, with intervals of 2138–5239 ms (3393 ± 1107), and emitted 10.80–24.64 calls per minute (16.61 ± 5.35). Calls were formed by one or two notes, each with 117–148 ms (130 ± 10) in duration, at intervals of 90–157 ms (123 ± 34 ms). In calls with two notes, first note had a dominant frequency (1.65 kHz) lower than the second note (1.89 kHz).

#### Distribution, natural history, conservation status, and extinction risk.

**Figure 7.**
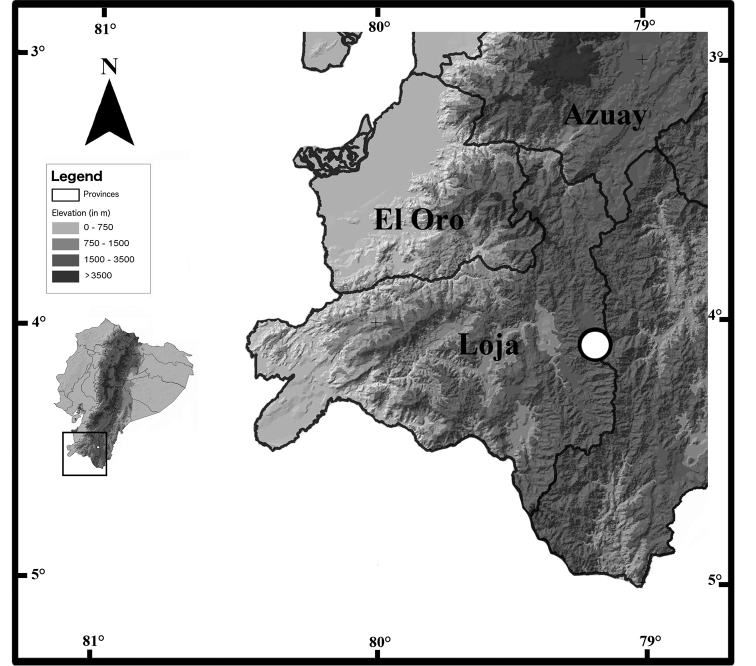
Map of southern Ecuador indicating the type locality of *Pristimantisandinogigas* sp. nov. at the Nudo de Cajanuma, Cordillera Oriental, Andes of southern Ecuador.

*Pristimantisandinogigas* sp. nov. is only known from its type locality, the paramos of the Nudo de Cajanuma, at elevations between 3300 and 3400 m, on the Cordillera Oriental of the Andes of southern Ecuador (Fig. 7). Surveys in other localities of the Nudo de Cajanuma, and on the nearby Nudo de Sabanilla, have not recorded the species (Almendáriz and Orcés 2004, Ron et al. 2019). However, most surveys were conducted at lower elevations, and most paramos in the region lack amphibian inventories. It is possible that *P.andinogigas* inhabits a larger area at the Cajanuma-Sabanilla mountain ridges; but it is unlikely that it occurs farther north on the Cordillera Oriental (e.g., Nudo de Guagrahuma), because of separation by the valley of the River Zamora, reaching elevations as low as 2800 m that may limit species’ dispersal.

The ecosystem at the type locality is Paramo Bambusoid Meadow (MAE et al. 2013). The most representative plant genera were *Bomarea*, *Miconia*, *Blechnum*, *Disterigna*, *Epidendrum*, *Gaultheria* and *Puya*; and the most abundant plant species were *Escalloniamyrtilloides*, *Puyanitida*, *Hypericumlancioides*, *Tillandsiaaequatorialis*, *Neurolepisnana*, *Cortaderiabifida*, *C.jubata*, *Chusqueaneurophylla*, *Calamagrostrismacrophylla*, *Themistoclesiaepiphytica*, *Seneciotephrosioides*, *Disterigmapentandrum*, and *D.empetrifolium*, *Rubuslaegaardii* (Eguiguren et al. 2015).

*Pristimantisandinogigas* was found active at night (19h00–22h00) at 6–10° C air temperature and 85–98% relative humidity. All males and some subadults and juveniles were observed active on bamboos (*Neurolepis* spp.); while both adult females were found active on the floor. During the day, individuals were found hidden inside rosettes (*Senecio* spp. and *Puya* spp.) or at the base of bamboos. *Pristimantisandinogigas* was the most abundant species during surveys at the type locality, representing 47 out of 108 anuran records. It was found in sympatry with *Pristimantispercultus*, Pristimantis sp. cf. colodactylus, Pristimantis sp. cf. orestes, and *Lynchus* sp.

The type locality of *P.andinogigas* is officially protected as part of the Podocarpus National Park, a national protected area created in 1982. The area has little anthropogenic impact, and in general, paramos of the Nudo de Cajanuma and the nearby Nudo de Sabanilla are reported to have a relatively good conservation status (Hofstede et al. 2002). Road infrastructure projects have been proposed in the past, but their development was cancelled (Cisneros et al. 2004, Bernardi de León 2009). However, three expeditions over the last five years have recorded very low numbers of *P.andinogigas*. Although more data are needed, it may be possible that the population of *P.andinogigas* has declined. In the absence of further information about the extinction risk on this newly discovered species, we suggest that *P.andinogigas* should be classified in the IUCN Red List category of Data Deficient (IUCN 2012).

## Discussion

*Pristimantisandinogigas* sp. nov. is morphologically similar to several species formerly associated under the *P.orcesi* species-group (i.e., *Pristimantiserythros*, *P.farisorum, P.obmutescens*, *P.orcesi*, *P.racemus*, *P.simoterus*, *P.simoteriscus*, and *P.thymelensis*). However, we refrain from assigning it to any species-group in the absence of data to conduct an integrative phylogenetic analysis. Morphological characters in *Pristimantis* are by themselves unreliable to assess phylogenetic affinities, and most of the species-groups within *Pristimantis* that were solely defined on morphology have resulted non-monophyletic (Pinto-Sánchez et al. 2012, Padial et al. 2014). The *Pristimantisorcesi* species-group proposed by Lynch (1981) was found to be non-monophyletic by Pinto-Sánchez et al. (2012) and Padial et al. (2014); although the relationships of most species remain unknown since both studies included only two species assigned to the *P.orcesi* species-group (*P.orcesi* and *P.thymelensis*).

*Pristimantisandinogigas* shows two notorious morphological characters that are not extended in the genus: glandular patches covering dorsal surfaces body and limbs, and porous dorsal skin texture. Similar glandular patches were first reported in P. *pycnodermis* by Lynch (1979), subsequently in *P.loujosti* by Yánez-Muñoz et al. (2010) and in *P.erythros* by Sánchez-Nivicela et al. (2018), and we have observed them in *P.orcesi* and an undescribed species of *Pristimantis* from the paramos of southern Ecuador. Porous skin texture has not been reported in any other species of *Pristimantis*, although it could have been confused with shagreen texture when not examined in detail or in preserved specimens. Further analyses are needed to understand the morphology of these characters and their phylogenetic significance.

Over the last decades, field studies in the Podocarpus National Park and nearby areas have revealed extraordinary flora and fauna diversity on the southernmost portion of the Cordillera Oriental of the Andes in Ecuador (Borchsenius 1997, Stattersfield et al. 1998, Brehm et al. 2005, Keating 2008, Rex et al. 2008, Richter et al. 2009). Although information on amphibians and reptiles has not been fully systematised and several areas remain unexplored, available data shows high levels of species richness and endemicity of amphibians and reptiles in the region (Lynch 1979, Cisneros-Heredia and McDiarmid 2006, Yánez-Muñoz et al. 2013, Torres-Carvajal et al. 2017). Further new species of amphibians and reptiles from the Podocarpus National Park, and the nearby Yacuri National Park, will be described in the near future, and discovery of additional new species from unexplored areas is expected.

## Supplementary Material

XML Treatment for Pristimantis andinogigas
